# Digital health solutions to improve health care: a call for papers

**DOI:** 10.2471/BLT.24.291451

**Published:** 2024-03-01

**Authors:** Divya Lakhotia, Rapeepong Suphanchaimat, Walaiporn Patcharanarumol, Alain Labrique, Viroj Tangcharoensathien

**Affiliations:** a International Health Policy Program, Ministry of Public Health, Tiwanon Road, Nonthaburi, 11000, Thailand.; b Department of Digital Health and Innovation, World Health Organization, Geneva, Switzerland.

Health inequities, within and across countries, stand as a critical global challenge of our time. Over 1 billion people face preventable diseases and premature deaths due to limited access to health care.^1^ The coronavirus disease 2019 (COVID-19) pandemic has further widened these disparities, with impacts disproportionately affecting people in vulnerable situations,^2^ highlighting the need to address the underlying structural inequality.^3^ Higher life expectancy and chronic noncommunicable disease prevalence have increased demand for health services, while declining fertility rates and adult workforce have decreased governments’ fiscal capacity and therefore financial allocations to the health sector.^4^ Inadequate government spending on health has increased out-of-pocket expenditure and financial hardship or unmet health-care needs for those who cannot afford to pay, with delayed access possibly resulting in disability or death.^5^

In our digitally interconnected world, capitalizing innovative technologies emerges as a promising approach to advance universal health coverage. More people have access to mobile phones than to sanitation facilities; in 2022, there were 108 mobile cellular subscriptions per 100 people worldwide,^6^ whereas only about 81% of the population was using at least basic sanitation services.^7^ This reality, while concerning, underscores the immense potential of digital health to reach a substantial portion of the global population. In 2021, the World Health Organization (WHO) developed the *Global strategy on digital health 2020–2025*.^8^ The strategy’s vision is to improve health for everyone, everywhere, by accelerating the development and adoption of appropriate, accessible, affordable, scalable and sustainable person-centric digital health solutions. Digital health solutions also aim to prevent, detect and respond to epidemics and pandemics, while also developing infrastructure and different types of applications that enable countries to use health data to promote health and well-being, and to achieve the health-related sustainable development goals and targets. This strategy provides useful guidance for countries in their efforts towards implementing a digital health policy and strategy.^8^

Digital health solutions can strengthen health systems and facilitate remote health-care services. For example, training the health workforce and providing teleconsultations to health workers and patients can make health care more accessible, equitable and affordable, especially for hard-to-reach marginalized populations and people in low- and middle-income countries.^9^ However, these digital solutions require interoperability of the existing health data. Using digital tools such as artificial intelligence for real-time remote diagnosis and treatment is poised to transform health-care provision due to these tools’ ability to unlock the potential of big data and fill the gap of skilled workforce shortages. For example, artificial intelligence applications can be used for diagnosis, health-care analytics and interpretation of magnetic resonance imaging and radiographs. Digital health is making inroads into nearly every facet of health system building blocks, ranging from training and education of the health workforce, health information systems, clinical decision support at the point of service to patient self-management of chronic conditions.^10^

The *Bulletin of the World Health Organization* calls for papers that provide evidence and experience on how the digital transformation in low- and middle-income countries has been applied in ensuring no one is left behind and that health-care services become more accessible – overcoming the digital divide – efficient, equitable and affordable. Papers should guide policy-makers and decision-makers on how to move forward in the digital transformation era, covering topics such as policy analyses on necessary enablers that accelerate digital transformation, notably standards, infrastructure, policy and governance; scalable solutions to reach marginalized and hard-to-reach populations; experiences in the shift towards a more digitalized health care in low- and middle-income countries; and evidence of how digital technologies have improved the efficiency, equity, accessibility and affordability of health care.

The *Bulletin* welcomes contributions from stakeholders, public health decision-makers, researchers and civil society and community representatives. Articles that address the impacts of health technologies and digital solutions on health inequities and affordability are welcome. We encourage manuscripts that identify effective, feasible, standardized and scalable use of health technologies.

The deadline for submissions is 16 June 2024. Manuscripts should be submitted in accordance with the *Bulletin*’s guidelines for contributors and the cover letter should mention this call for papers. 
